# NEAT1 Coordinates a PDLIM5–CACNA1C Regulatory Program Associated with a Potentially Arrhythmogenic Cardiomyocyte State in the Border Zone During Early Myocardial Infarction

**DOI:** 10.3390/ijms27177945

**Published:** 2026-09-07

**Authors:** Jiuxiao Zhao, Qian Zhu, Yang Lou, Mingmin Zhou, Yameng Chen, Shiquan Chen, Qiang Liu, Chenyang Jiang

**Affiliations:** 1 Center for Cardiac Arrhythmias and Cardiomyopathy (Clinical & Research), Sir Run Run Shaw Hospital (SRRSH-CAC), Hangzhou 310016, China; 3190100251@zju.edu.cn (J.Z.); zhuqian427@zju.edu.cn (Q.Z.);; 2 Department of Veterinary Medicine, College of Animal Sciences, Zhejiang University, Hangzhou 310058, China; 3 Department of Cardiology, Sir Run Run Shaw Hospital, Zhejiang University School of Medicine, Hangzhou 310016, China; 4 Zhejiang Key Laboratory of Cardiovascular Intervention and Precision Medicine, Hangzhou 310016, China; 5 Engineering Research Center for Cardiovascular Innovative Devices of Zhejiang Province, Hangzhou 310016, China

**Keywords:** myocardial infarction, single-cell transcriptomics, cardiomyocyte heterogeneity, non-coding RNA, in silico knockout

## Abstract

Patients with early-stage myocardial infarction (MI) are at high risk of malignant ventricular arrhythmias, yet the cell-type-specific molecular landscape associated with post-infarction arrhythmogenesis has not been systematically characterized. This study integrates single-nucleus and spatial transcriptomics to define a cardiomyocyte subpopulation in early MI and dissect the *NEAT1*-centered regulatory network driving its ion channel remodeling. Single-nucleus transcriptomic data from post-MI human hearts were re-analyzed to identify a distinct subpopulation, termed arrhythmia-potential cardiomyocytes (aCMs), within the infarct border zone, characterized by pronounced ion channel remodeling. Gene co-expression network analysis revealed two modules highly associated with aCMs, in which NEAT1 correlated with the calcium channel gene *CACNA1C* and the LIM domain protein PDLIM5. All three genes were upregulated in hypoxic rat cardiomyocytes; siRNA-mediated knockdown confirmed that *NEAT1* silencing downregulated *CACNA1C* and *PDLIM5* expression, consistent with in silico knockout predictions. A ceRNA network further identified *hsa-miR-204-5p/211-5p* as a key mediator consistent with regulatory axis. These findings suggest that cardiomyocytes in the early MI border zone exhibit ion channel remodeling driven by elevated *NEAT1*, which may modulate *CACNA1C* and *PDLIM5* through a microRNA-mediated ceRNA network, suggesting that targeting *NEAT1* may warrant further investigation for preventing malignant arrhythmias in early-stage MI.

## 1. Introduction

Myocardial infarction (MI) remains a leading cause of mortality worldwide, with malignant ventricular arrhythmias representing the predominant mechanism of sudden cardiac death in the acute phase [1]. Clinical studies have consistently demonstrated that patients with early-stage MI are particularly vulnerable to life-threatening arrhythmias [1]. Up to 90% of patients with acute MI develop some form of arrhythmia during or immediately after the event, with the majority occurring within the first 24 to 48 h [2]. The initial 5 days post-MI are recognized as the early phase of infarction, during which the risk of arrhythmias remains elevated [3]. Ventricular fibrillation may occur without prior warning arrhythmias, especially during the first day of infarction [4]. Collectively, these observations underscore that the early post-infarction period represents a critical window of arrhythmia susceptibility [5,6,7]. Despite advances in reperfusion therapy, malignant ventricular arrhythmias remain a major early complication after ST-segment elevation MI [8], underscoring an unmet need for mechanism-based preventive strategies.

At the cellular and molecular levels, post-infarction arrhythmogenesis has been associated with several discrete pathological processes. These include ion channel remodeling, such as dysregulation of *CACNA1C*, *SCN5A*, and *KCNQ1*, gap junction dysfunction mediated by connexin 43 (Cx43) post-translational modifications [9], aberrant calcium handling involving CaMKII activation [10], and myofibroblast accumulation within the infarct border zone, which creates a substrate for slow conduction and re-entry [11,12]. However, these mechanisms have largely been investigated in isolation and at the bulk-tissue level, leaving the cell-type-specific molecular landscape that governs arrhythmia susceptibility in early-stage MI incompletely characterized.

Recent advances in single-cell and spatial transcriptomics have enabled unbiased, high-resolution dissection of cellular heterogeneity in the diseased heart. Single-nucleus RNA sequencing (snRNA-seq) has been successfully applied to delineate the cellular dynamics of post-MI repair and remodeling [13,14], and spatial transcriptomic analyses have begun to map the molecular architecture of the infarct border zone [14,15]. However, these approaches have yet to be systematically leveraged to investigate the molecular basis of arrhythmia susceptibility in the early post-MI period.

In this study, by integrating single-cell and spatial transcriptomic analyses of post-infarction human hearts, we identify a border-zone-localized cardiomyocyte subpopulation characterized by pronounced ion channel remodeling. Integrative network analyses and functional validation further pinpoint the long non-coding RNA *NEAT1* as a key regulator of calcium homeostasis within this subpopulation, suggesting *NEAT1* as a potential target for post-infarction arrhythmias.

## 2. Results

### 2.1. Identification of Arrhythmia-Potential Cardiomyocytes in Early-Stage MI

**Figure 1:**
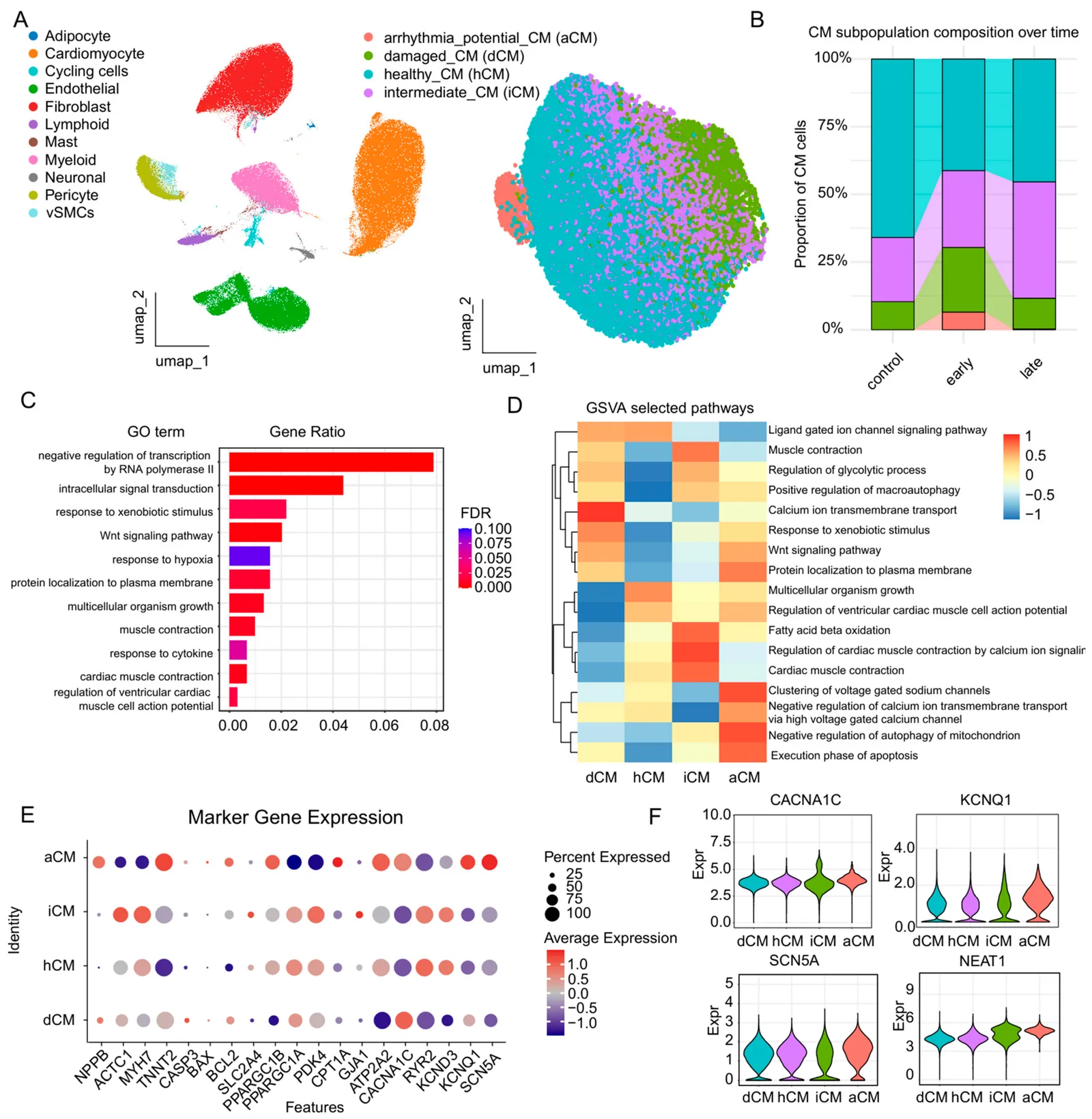
Identification of arrhythmia-potential cardiomyocytes in early-stage MI. (**A**) UMAP visualization of snRNA-seq data from all samples (left; *n* = 191,795) and cardiomyocytes (right; *n* = 55,204). (**B**) Bar plot depicting the proportions of cardiomyocyte subtypes at early (infarction duration < 5 days) versus late (infarction duration > 5 days) stages. (**C**) Bar plot of GO enrichment analysis for differentially expressed genes between aCMs and hCMs; the *x*-axis represents gene ratio and color indicates FDR. (**D**) Heatmap showing GSVA enrichment scores across cardiomyocyte subtypes. (**E**) Dot plot displaying expression levels of marker genes across cardiomyocyte subtypes. (**F**) Violin plot showing expression levels of ion channel genes across cardiomyocyte subtypes. Data are shown as mean ± SD.

Through re-analysis of snRNA-seq data from human hearts following MI, we defined a distinct cardiomyocyte subpopulation (Figure 1A), termed arrhythmia-potential cardiomyocytes (aCMs). Patients were stratified into early-stage MI (≤5 days post-infarction) and late-stage MI (>5 days) based on the time from symptom onset. Cell proportion analysis revealed that aCMs were predominantly enriched in early-stage MI samples and were nearly absent in late-stage MI and control hearts (Figure 1B). To characterize the transcriptional features of this subpopulation, we compared aCMs with healthy cardiomyocytes. Gene Ontology (GO) analysis of differentially expressed genes (DEGs) showed significant enrichment in pathways related to cardiac muscle contraction and the regulation of ventricular cardiac muscle cell action potential (Figure 1C). We further performed gene set variation analysis (GSVA) across the identified cardiomyocyte subpopulations (Figure 1D). Compared with damaged cardiomyocytes (damage_CMs), aCMs exhibited preserved fatty acid β-oxidation capacity, while multiple ion channel-related pathways were markedly altered, indicative of ion channel remodeling. Consistently, aCMs showed elevated expression of key ion channel genes, including *KCNQ1*, *CACNA1C*, and *SCN5A* (Figure 1E,F). Notably, while *PPARGC1A* expression was lower in aCMs, *PPARGC1B* remained relatively high (Figure 1E and Figure S1A).

To assess whether the aCM cell state is generalizable rather than cohort-specific, we re-analyzed an independent human single-cell dataset comprising one early-MI heart (5 days post-infarction) [16] and one control donor. UCell scoring against the aCM signature identified clusters 2 with markedly elevated aCM scores, whereas cluster 1, dominated by control cardiomyocytes, showed high hCM scores (Figure S2C). Violin plots of representative aCM signature genes further confirmed that clusters 2 recapitulated the aCM transcriptional program (Figure S2D). These results support the existence and robustness of aCMs in an independent early-MI sample.

Collectively, these results define a new cardiomyocyte subpopulation present in the heart during early-stage MI, characterized by pronounced ion channel remodeling and sustained fatty acid metabolism, potentially representing a stress-responsive cardiomyocyte state of therapeutic relevance.

### 2.2. Arrhythmia-Potential Cardiomyocytes Localize to the Infarct Border Zone in Early-Stage MI

**Figure 2:**
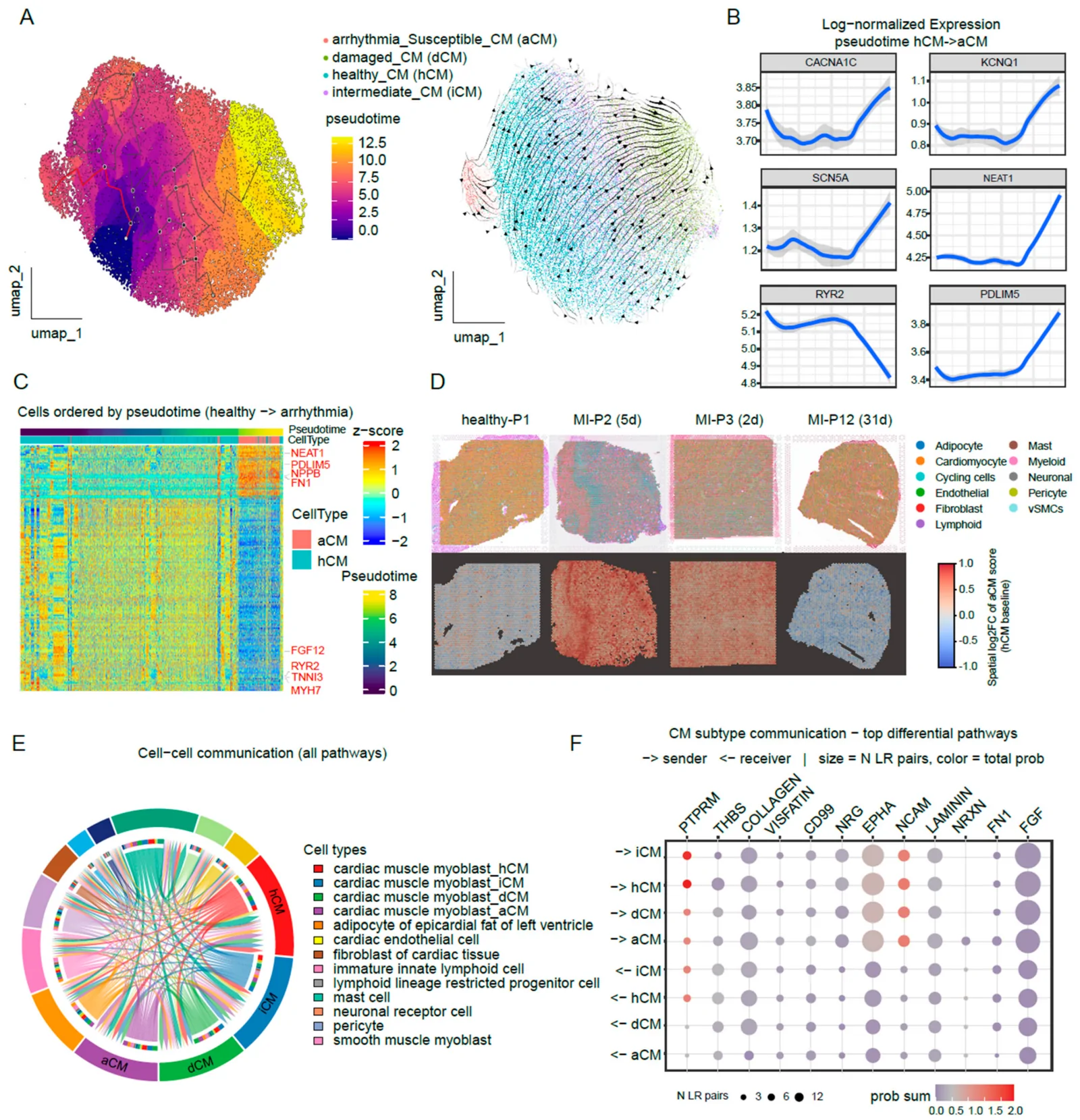
Pseudotime inference, spatial profiling, and intercellular communication analysis of arrhythmogenic cardiomyocytes. (**A**) Pseudotime trajectory analysis of cardiomyocyte subtypes (left) and scTour RNA velocity analysis (right); the red curve indicates the differentiation trajectory toward aCMs. (**B**) Expression curves of ion channel-related genes along the hCM-to-aCM pseudotime trajectory (log-normalized expression; pseudotime: hCM to aCM). (**C**) Heatmap showing the expression of a subset of differentially regulated genes along the hCM to aCM trajectory, identified by Monocle 3. (**D**) Upper: H&E-stained tissue sections overlaid with pie charts depicting cell type composition at individual spatial spots; lower: normalized aCM score. (**E**) Chord diagram illustrating outgoing and incoming signaling among all cell types, with cardiomyocytes further resolved into subtypes. (**F**) Dot plot displaying the top 10 differentially enriched intercellular communication pathways between aCMs and hCMs; dot size represents number of ligands–receptor pairs, and color indicates communication probability.

Given that pseudotime analysis can reconstruct cell-state transitions during pathological remodeling [17], we applied Monocle3 to order aCMs within the cardiomyocyte differentiation landscape. Pseudotime ordering positioned aCMs between hCMs and iCMs (Figure 2A). We subsequently conducted RNA velocity analysis using scTour, which revealed that while cardiomyocytes in MI hearts predominantly followed an hCM→iCM→dCM trajectory, aCMs appeared to diverge from this canonical progression (Figure 2A). We extracted cells along the hCM-to-aCM path, visualized their transcriptional dynamics by heatmap, and profiled the expression changes in ion channel genes along pseudotime. Notably, ion channel gene expression increased sharply with advancing pseudotime (Figure 2B,C). Collectively, pseudotime and RNA velocity analyses suggest that aCMs follow a distinct differentiation trajectory and that ion channel remodeling represents a defining feature of their development.

To determine the spatial localization of aCMs in the post-MI heart, we re-analyzed spatial transcriptomic data from the infarct border zone of control, two early-stage MI, and one late-stage MI samples. aCM abundance in each Visium spot was estimated by scoring aCM signature derived from reference scRNA-seq data (Wilcoxon test, adjusted *p* < 0.01, log_2_FC > 1.0) and normalizing across all CM subclusters via softmax. Consistent with pseudotime findings, aCMs were almost exclusively detected in early-stage MI hearts. Moreover, aCM proportions were slightly elevated in regions with higher fibroblast abundance (Figure 2D). Overall, spatial transcriptomic analysis corroborated our pseudotime results, confirming that aCMs predominantly reside in the infarct border zone of early-stage MI.

Finally, to characterize the cell–cell communication landscape of aCMs, we performed ligand–receptor interaction analysis using CellChat.Chord diagram analysis revealed that the overall communication strength of aCMs was slightly lower than that of hCMs (Figure 2E), but comparable to that of iCMs and dCMs. Differential pathway analysis between aCMs and hCMs, followed by dot plot visualization of the top 10 altered pathways, showed that signaling through the cell adhesion-related PTPRM pathway and the extracellular matrix-related THBS pathway was decreased in aCMs, whereas signaling through the synaptic-related NRXN pathway and the fibroblast extracellular matrix-related FN1 pathway was increased (Figure 2F). Consistently, the increased FN1-related signaling in aCMs aligned with our spatial transcriptomic observation that aCMs were preferentially localized to fibroblast-rich regions.

### 2.3. Construction of Weighted Co-Expression Network and Identification of aCM-Related Key Modules

**Figure 3:**
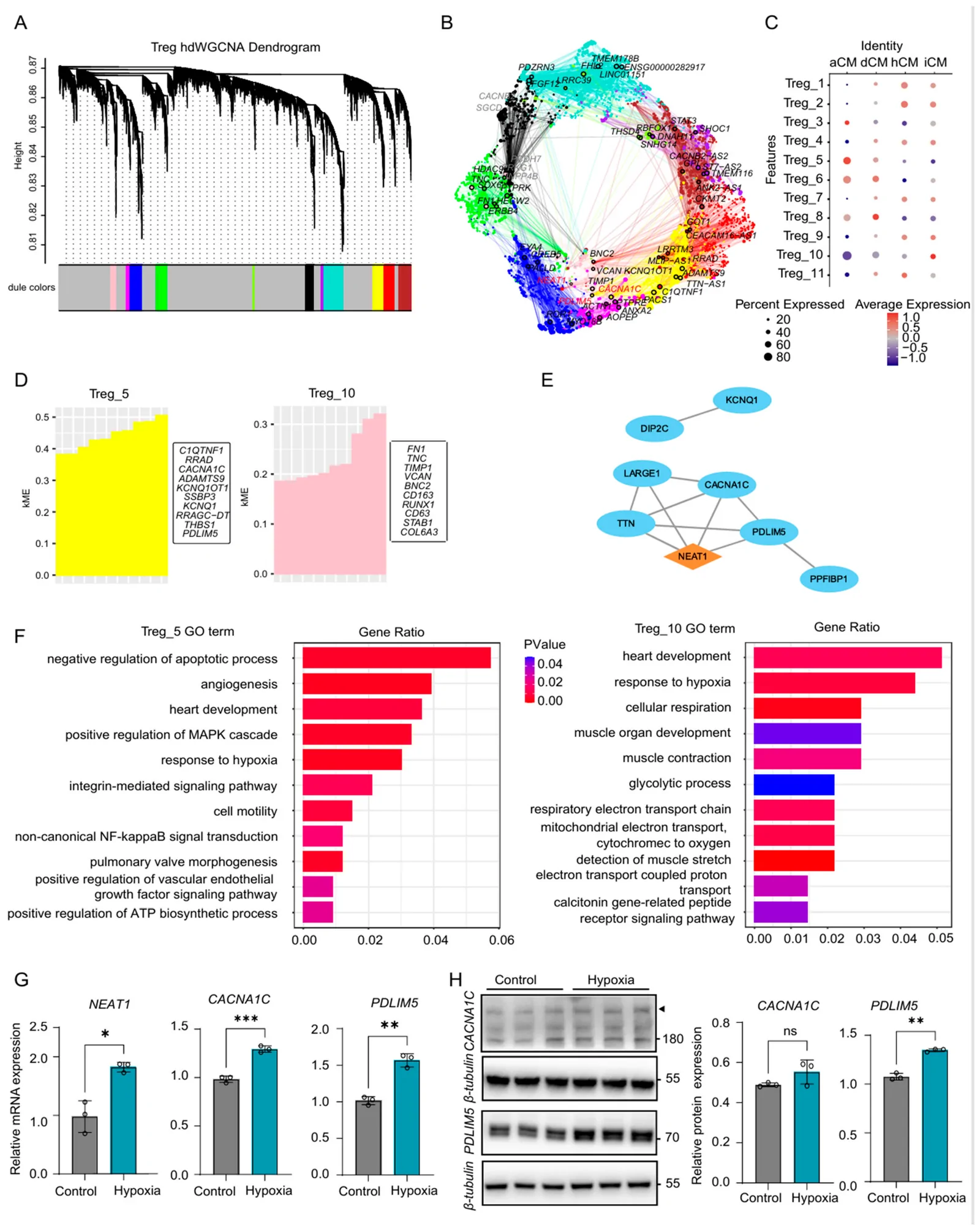
Co-expression network construction and identification of key regulatory modules in aCMs. (**A**) Cluster dendrogram of genes in the hdWGCNA co-expression network, with branches colored by module assignment. (**B**) UMAP visualization of the hdWGCNA gene co-expression network; each point represents a gene, colored by module membership. (**C**) Dot plot showing expression patterns of genes from different modules across cardiomyocyte subtypes; dot size indicates the proportion of cells expressing a given gene, and color represents average expression level. (**D**) Bar plot displaying the top 10 hub genes (ranked by kME) of module 5 and module 10. (**E**) Regulatory network constructed from highly correlated genes; blue ellipses denote mRNAs and orange diamonds denote lncRNAs. (**F**) Bar plot of GO enrichment analysis for genes in module 5 and module 10; the *x*-axis represents gene ratio, and color indicates the *p* value. (**G**) qPCR analysis of NEAT1, CACNA1C, and PDLIM5 transcript levels in NRVMs following 6 h of hypoxia treatment. (**H**) Representative Western blot images (left) and quantification (right) of CACNA1C and PDLIM5 protein levels in NRVMs following 6 h of hypoxia treatment. The black arrowhead denotes CACNA1C (~240 kDa), with molecular size estimated against the 180 kDa protein marker. Data are shown as mean ± SD. * *p* < 0.05, ** *p* < 0.01, *** *p* < 0.001.

To identify transcriptional drivers of aCM-specific ion channel remodeling, we performed hdWGCNA on aCM-centered single-cell transcriptomes. This analysis yielded 11 co-expression modules, ranging from 67 to 667 genes per module (Figure 3A and Figure S1B). Module eigengene profiling across cell types revealed that two modules, Module 5 and Module 10, were most differentially expressed in aCMs, with M5 significantly elevated and M10 markedly depleted relative to other cardiomyocyte subsets (Figure 3C). UMAP embedding of module gene sets confirmed their spatial proximity in the transcriptional landscape (Figure 3B). We therefore prioritized these two modules for downstream analysis. The core genes of Module 5 are mainly involved in ion channel activity and extracellular matrix interactions, including *CACNA1C*, *KCNQ1*, and *THBS1*, while those of Module 10 are mainly involved in extracellular matrix remodeling and inflammatory responses, including *FN1*, *TIMP1*, and *CD163* (Figure 3D). GO enrichment analysis demonstrated that Module 5 genes were predominantly associated with signal transduction, such as MAPK, integrin, VEGF, and NF-κB, angiogenesis, anti-apoptosis, and ATP biosynthesis, whereas Module 10 genes converged on energy metabolism, including respiration, glycolysis, and electron transport, and muscle contraction (Figure 3F). Notably, both modules were commonly enriched in heart development and response to hypoxia.

To identify key regulatory interactions, we performed pairwise correlation analysis within the two modules, retaining only edges with correlation coefficients >0.75 to construct a regulatory network (Figure 3E). Network analysis revealed that the calcium channel gene *CACNA1C* was under the regulation of multiple genes, among which two emerged as prominent nodes: the long non-coding RNA *NEAT1* and *PDLIM5*, which were among the most highly correlated gene pairs in the network.

We next validated these findings using a neonatal rat ventricular myocyte (NRVM) hypoxia model. Consistent with the single-cell data, both *Neat1* *Pdlim5* and *Cacna1c* transcript levels were significantly upregulated following 6 h of hypoxia exposure. At the protein level, however, PDLIM5 expression was markedly increased, whereas CACNA1C showed no significant change under the same conditions (Figure 3G,H). Given that *NEAT1* ranks among the most prominent marker genes of aCMs, these results suggest that *NEAT1* may act as a master regulator driving the arrhythmia-potential phenotype of aCMs, potentially through modulation of calcium channel-associated genes *CACNA1C* and *PDLIM5*.

### 2.4. NEAT1 Regulates CACNA1C and PDLIM5 Expression

**Figure 4:**
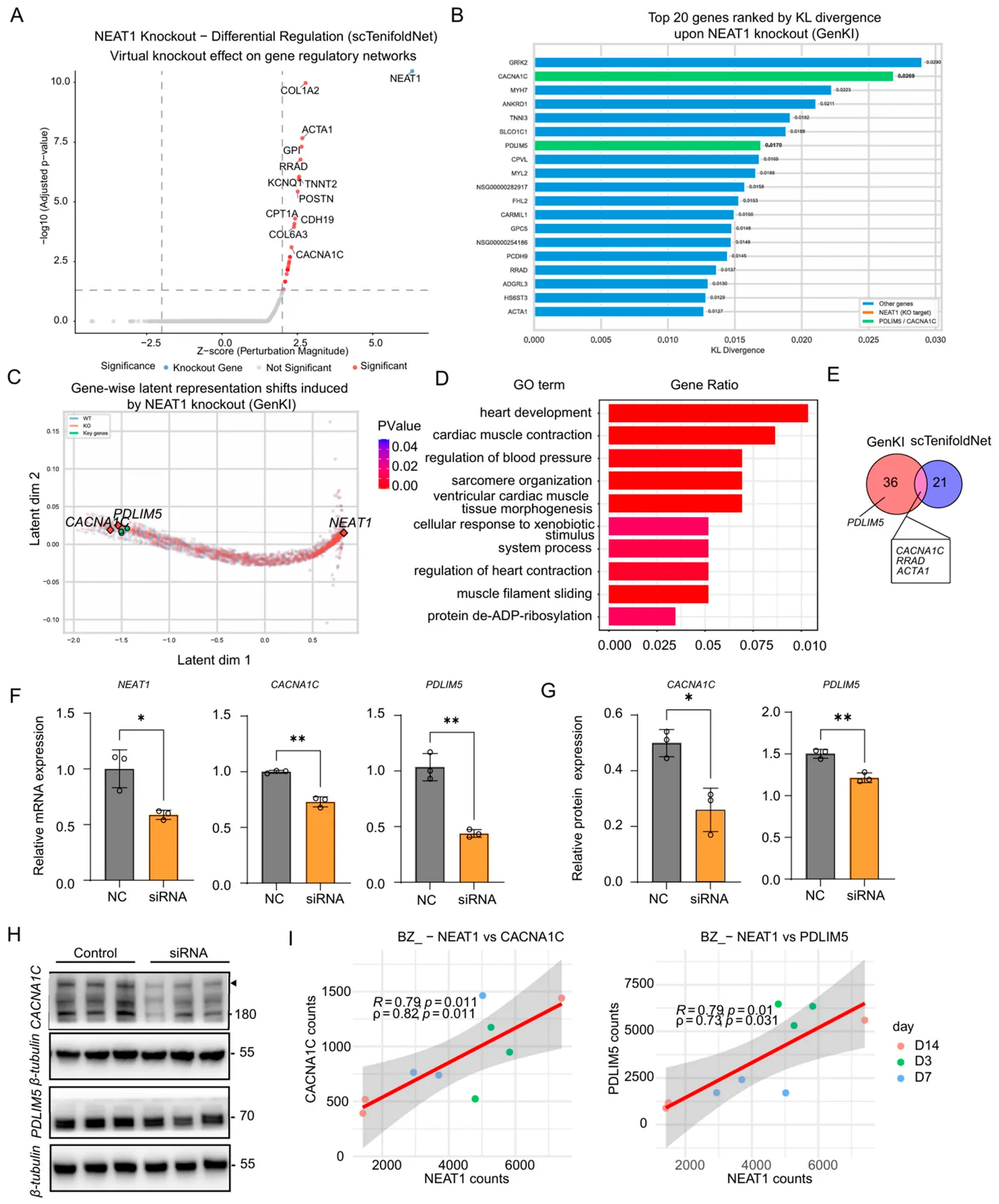
Virtual knockout and siRNA-mediated knockdown validation of *NEAT1* regulation of *CACNA1C* and *PDLIM5*. (**A**) Volcano plot displaying the results of scTenifoldNet virtual knockout of NEAT1. (**B**) Bar plot showing the top-ranked genes most significantly affected upon NEAT1 knockout, ranked by KL divergence; longer bars indicate greater shifts in expression distribution. CACNA1C (rank 3) and PDLIM5 (rank 8) are highlighted in green. (**C**) Latent space visualization of gene expression shifts from WT to KO. Gray lines represent displacement vectors of the top 30 significantly perturbed genes, with longer lines indicating larger shifts. Blue and red scatter points denote the background distributions of all genes under WT and KO conditions, respectively. Green circles and red diamonds mark the WT and KO positions of NEAT1, PDLIM5, and CACNA1C. (**D**) Bar plot of GO enrichment analysis for significantly perturbed genes predicted by scTenifoldNet and GeneKI; the *x*-axis represents gene ratio, and color indicates FDR. (**E**) Venn diagram showing the overlap of significantly perturbed genes predicted by scTenifoldNet and GeneKI. (**F**) qPCR analysis of Neat1, Cacna1c, and Pdlim5 transcript levels in NRVMs following siRNA-mediated knockdown of NEAT1. (**G**) Quantification of Cacna1c and Pdlim5 protein levels by Western blot in NRVMs following siRNA-mediated knockdown of NEAT1. (**H**) Representative Western blot images of CACNA1C and PDLIM5 protein levels in NRVMs following siRNA-mediated knockdown of NEAT1. (**I**) Validation of NEAT1–CACNA1C and NEAT1–PDLIM5 correlations in the GSE110209 dataset. The black arrowhead denotes CACNA1C (~240 kDa), with molecular size estimated against the 180 kDa protein marker. Data are shown as mean ± SD. * *p* < 0.05, ** *p* < 0.01.

To assess the robustness of the hdWGCNA-derived prediction, we first performed in silico knockout experiments using two distinct computational approaches, scTenifoldNet and GeneKI. scTenifoldNet simulated NEAT1 perturbation by comparing wild-type and knockout network topologies, revealing a pronounced impact on *CACNA1C* (|Z| > 2, adjusted *p* < 0.05) (Figure 4A). GeneKI applied a variational graph auto-encoder (VGAE) to predict the downstream consequences of *NEAT1* depletion, identifying 39 significantly perturbed genes (Figure 4B), among which *CACNA1C* ranked 3rd (KL divergence = 0.027, hit = 100/100) and *PDLIM5* ranked 8th (KL divergence = 0.017, hit = 100/100), confirming that both genes are strongly and consistently affected by *NEAT1* depletion. GO analysis of the genes predicted to be significantly altered upon *NEAT1* depletion indicated predominant involvement in myocardial contraction, sarcomere organization, and blood pressure regulation, further supporting the functional relevance of this regulatory axis (Figure 4D).

We next experimentally validated these predictions by siRNA-mediated knockdown of *NEAT1* in NRVMs. qPCR and Western blot analyses confirmed that *NEAT1* silencing significantly downregulated both *CACNA1C* and *PDLIM5* at the transcript and protein levels (Figure 4F–H), consistent with the in silico predictions. As corroborative evidence, we re-analyzed publicly available transcriptomic data from the mouse infarct border zone across post-MI time points (3, 7, and 14 days). *Neat1*, *Cacna1c*, and *Pdlim5* expression peaked at day 3 and progressively declined at days 7 and 14, mirroring the temporal window of heightened arrhythmia susceptibility (Figure 4I). Moreover, their expression was tightly correlated in the post-MI border zone but not in control hearts (Figure S1C).

Taken together, our siRNA-mediated knockdown experiments provide direct causal evidence that *NEAT1* regulates *CACNA1C* and *PDLIM5* expression, which is further supported by in silico perturbation and in vivo transcriptomic analyses.

### 2.5. NEAT1 Knockdown Identifies hsa-miR-204-5p/211-5p as a Potential Mediator of CACNA1C and PDLIM5 Regulation

**Figure 5:**
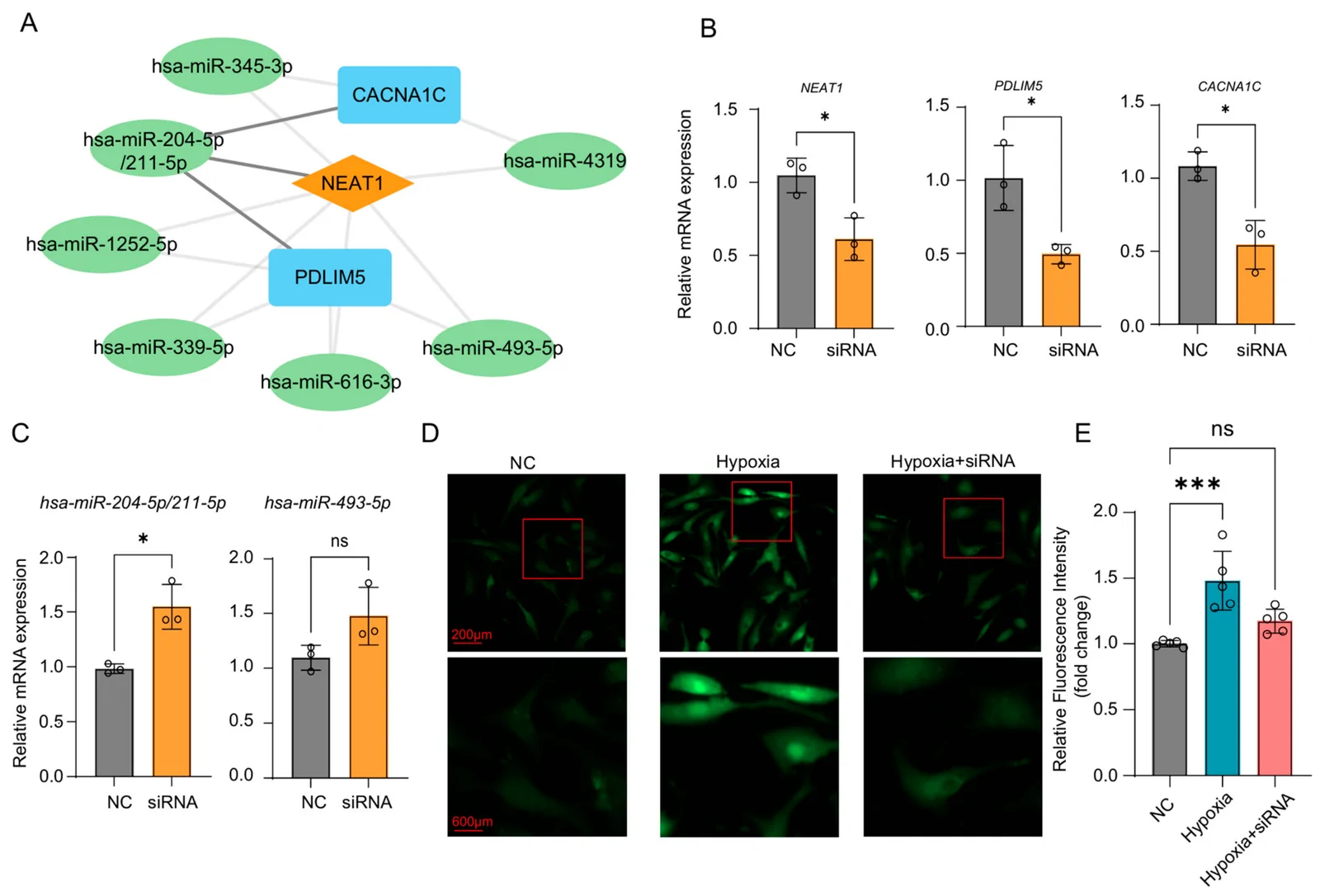
*NEAT1* regulates *CACNA1C* and *PDLIM5* through a microRNA-mediated ceRNA mechanism. (**A**) ceRNA regulatory network constructed based on database-predicted interactions. Blue ellipses denote mRNAs, orange diamonds represent lncRNAs, and green ellipses indicate microRNAs. (**B**) qPCR analysis of *NEAT1*, *CACNA1C*, and *PDLIM5* transcript levels in AC16 cells following siRNA-mediated knockdown of *NEAT1* (**C**) qPCR analysis of *hsa-miR-204-5p/211-5p* and *hsa-miR-493-5p* transcript levels in AC16 cells following siRNA-mediated knockdown of *NEAT1*. (**D**) Fluo-4 AM staining detected intracellular Ca^2+^ levels in NC, Hypoxia, and Hypoxia + siRNA-mediated knockdown of *NEAT1* groups. Green fluorescence intensity reflects the relative cytosolic calcium concentration. Scale bars: 600 µm (upper panels) and 200 µm (lower panels, higher-magnification views of the corresponding upper panels). (**E**) Quantification of Fluo-4 fluorescence intensity in NC, Hypoxia, and Hypoxia + siRNA-mediated knockdown of *NEAT1* groups. Data are shown as mean ± SD. * *p* < 0.05, *** *p* < 0.001.

Given that lncRNAs frequently act as competing endogenous RNAs (ceRNAs) by sequestering microRNAs, we screened for candidate microRNAs targeting *NEAT1*, *CACNA1C*, and *PDLIM5* using two independent databases, Starbase and ENCORI (Figure 5A). This analysis identified *hsa-miR-204-5p/211-5p* as a shared candidate. In AC16 human cardiomyocytes, siRNA-mediated *NEAT1* knockdown significantly upregulated *hsa-miR-204-5p/211-5p* while concomitantly reducing *CACNA1C* and *PDLIM5* expression (Figure 5B,C), a pattern consistent with a ceRNA-mediated regulatory relationship. Additionally, we examined the expression of other cardiac-related microRNAs that were predicted to be involved in the network. Most of these microRNAs did not show statistically significant changes upon *NEAT1* silencing. However, several microRNAs, including *hsa-miR-493-5p* and *hsa-miR-1252-5p* [18,19] (Figure 5C,D), exhibited an upward trend, suggesting that they might be subject to secondary regulatory influences beyond the primary ceRNA axis.

To functionally probe this axis, we performed Fluo-4 calcium imaging in hypoxia-exposed NRVMs. *NEAT1* knockdown attenuated hypoxia-induced elevation in intracellular Ca^2+^ levels (Figure 5D,E), suggesting a potential functional relevance of this regulatory axis in the ischemic myocardium.

## 3. Discussion

Ion channel remodeling is recognized as a central substrate for post-infarction arrhythmogenesis [20]; however, the cell-type-specific transcriptional programs driving this process remain poorly defined, particularly at the resolution of discrete cardiomyocyte subpopulations. Here, we analyzed single-nucleus and spatial transcriptomic data from human hearts following MI, and defined a distinct cardiomyocyte subpopulation, arrhythmia-potential cardiomyocytes (aCMs), localized to the infarct border zone. These aCMs exhibit pronounced ion channel remodeling with relatively preserved metabolic activity, and pseudotime and RNA velocity analyses revealed their unique trajectory intermediate between healthy and damaged CMs. Co-expression network analysis prioritized *NEAT1* as a hub gene strongly correlated with the L-type calcium channel gene *CACNA1C* and the L-type calcium channel regulator *PDLIM5*. Functional validation demonstrated that *NEAT1* positively regulates both targets likely through a microRNA-mediated ceRNA network. These findings identify *NEAT1* as a previously unrecognized regulator of ion channel gene expression in aCMs and suggest that targeting the *NEAT1*–*hsa-miR-204-5p/211-5p*–*CACNA1C*/*PDLIM5* axis may represent a candidate therapeutic target for arrhythmia prevention in early-stage MI.

By performing further clustering and subpopulation analysis, we defined a distinct subpopulation within hCMs, which we designated as aCMs. We defined early MI as occurring within five days of infarction onset. This definition is consistent with the well-established temporal phases of post-infarction cardiac repair, in which the acute inflammatory phase predominates during the first 4–5 days after MI before transitioning to the proliferative and maturation phases [21,22]. Cell proportion analysis revealed that aCMs were almost exclusively detected in the hearts of patients with early MI. GO enrichment analyses demonstrated that aCM marker genes were prominently clustered in pathways such as “response to hypoxia,” distinguishing them clearly from healthy hCMs. GSVA analysis found that aCMs retained a certain level of fatty acid β-oxidation activity, whereas pathways related to ion channel function were markedly altered. Notably, the pathway “negative regulation of calcium ion transmembrane transport via high voltage-gated calcium channel” was significantly upregulated, despite a concurrent increase in *CACNA1C* expression. This apparent paradox, in which enhanced channel transcription coincided with activated inhibitory regulation, may suggest a potential compensatory feedback mechanism that possibly reflect a transitional state of ion channel remodeling [23,24]. Gene-level analysis revealed that *PPARGC1A* was markedly downregulated in aCMs, whereas *PPARGC1B* was notably upregulated, a reciprocal pattern that may explain the preserved fatty acid metabolic capacity. In contrast, violin plots showed that both *PPARGC1A* and *PPARGC1B* expression were dysregulated in dCMs. Furthermore, aCMs highly expressed the potassium channel gene *KCNQ1*, the calcium channel gene *CACNA1C*, and the sodium channel gene *SCN5A*, collectively indicating a transcriptional signature consistent with electrical remodeling. This transcriptional program was further recapitulated in an independent single-cell dataset, in which aCM-like subclusters displayed high aCM signature scores, supporting the generalizability of the aCM phenotype beyond the discovery cohort.

To delineate the temporal trajectory of aCMs during post-MI cardiac remodeling, we performed pseudotime analysis and RNA velocity analysis. Notably, aCMs appeared to diverge from the canonical hCM–ICM–dCM disease progression axis. By examining marker genes along the trajectory from hCMs to aCMs, we found that *FN1*, *NEAT1*, *PDLIM5*, and *NPPB* were significantly upregulated during this transition. *FN1* encodes fibronectin, a key mediator of cell adhesion, and is closely associated with fibrotic processes [25,26]. We therefore interrogated our spatial transcriptomics data. Based on an aCM gene signature score, we found that aCMs were predominantly localized to the border zone of hearts from patients with early MI, whereas they were rarely detected in hearts from patients with late MI. Additionally, aCMs exhibited partial spatial colocalization with fibroblasts. Finally, to characterize the communication features of aCMs, we performed CellChat analysis. Chord diagram analysis revealed that both outgoing and incoming signaling of aCMs were slightly reduced compared with hCMs; in particular, the PTPRM signaling pathway was markedly attenuated, which may represent an important contributor to the impaired contractile function of aCMs [27]. Consistent with the spatial transcriptomics findings, the NRXN and FN1 signaling pathways were enhanced in aCMs, suggesting intensified crosstalk with fibroblasts. Notably, NRXN has also been implicated in calcium signaling [28].

Gene co-expression networks in biological systems typically follow a scale-free topology, endowing them with both resilience to random perturbations and vulnerability to targeted disruption of hub nodes [29]. We therefore applied hdWGCNA to identify the regulatory networks underlying aCMs. Among the 11 identified co-expression modules, module 5 and module 10 accounted for relatively high expression proportions. Notably, module 5 exhibited the highest average expression level among all modules, whereas module 10 exhibited the lowest, yet the two modules were closely adjacent in UMAP space. They were therefore selected as the two primary modules for downstream analysis. To identify the core regulatory network, we merged genes from modules 5 and 10, performed correlation analysis, and retained edges with a correlation coefficient greater than 0.75. We found that *KCNQ1* and *DIP2C* may form a regulatory module. Although *DIP2C* has been implicated in diverse molecular pathways, its regulatory relationship with MI and *KCNQ1* remains poorly characterized [30]. In addition, a larger regulatory network centered on *CACNA1C* was identified, encompassing *NEAT1*, *PDLIM5*, *TTN*, and *LARGE1*, among others. *NEAT1* has been reported to be upregulated after MI and to impair cardiac function [31]. Moreover, *NEAT1* has recently been shown to promote pathological cardiac remodeling and heart failure by driving paraspeckle formation through liquid–liquid phase separation, which sequesters Fth1 mRNA and triggers cardiomyocyte ferroptosis [32], further supporting a pathogenic role of *NEAT1* in the stressed myocardium. *PDLIM5* has been shown to regulate L-type calcium channel (Cav1.2) activity by forming a complex with protein kinase D1 (PKD1) [33]. We validated these transcriptional changes using an NRVM model exposed to 6 h of hypoxia. At the transcriptional level, 6 h of hypoxia significantly upregulated the expression of *Neat1*, *Cacna1c*, and *Pdlim5*. At the protein level, however, only PDLIM5 showed a significant increase.

Although the dynamics of *CACNA1C* in the border zone during early MI have not been definitively established, Wang et al. reported Cav1.2 protein downregulation at 12 h post-MI in mice [34], whereas Perrier E et al. [23] showed Cav1.2 mRNA upregulation at 1 week post-MI via mineralocorticoid receptor activation in rats. Beyond these model- and species-specific differences, the dissociation between *CACNA1C* mRNA and protein levels merits a more direct mechanistic consideration. First, the α1C subunit is a large multi-pass membrane protein (approximately 220 kDa, 24 transmembrane segments), and the synthesis, folding, membrane trafficking, and degradation of such multi-pass channel proteins occur over relatively long timescales [35]; within the acute 6 h window of our experiment, hypoxia-induced transcriptional upregulation may therefore not yet be translated into a measurable increase in Cav1.2 protein, whereas the smaller *PDLIM5* protein may respond within this period. Second, steady-state channel abundance is buffered at the post-transcriptional level, including microRNA-mediated translational repression and ubiquitin-proteasome-mediated degradation, which can maintain Cav1.2 protein despite elevated mRNA [36,37]. Notably, the *NEAT1*-*hsa-miR-204-5p/211-5p*-*CACNA1C*/*PDLIM5* ceRNA axis identified in this study operates precisely at this post-transcriptional layer. Third, the combined hypoxia and knockdown data suggest a necessary-but-not-sufficient relationship: *NEAT1* silencing was sufficient to reduce both *CACNA1C* mRNA and protein, whereas *NEAT1* upregulation alone may be insufficient to drive an increase in steady-state Cav1.2 protein within the experimental window. Finally, the lack of CACNA1C protein change in our hypoxia model may also reflect the acute in vitro conditions and the border-zone-specific aCM microenvironment, which a cell-based model cannot fully replicate.

We then employed two virtual knockout approaches, scTenifoldNet [38] and GeneKI [39], as internal robustness checks on the *NEAT1*–*CACNA1C*/*PDLIM5* regulatory relationship identified by hdWGCNA. Both approaches predicted that *NEAT1* knockout would perturb ion channel and contractile apparatus genes, including *CACNA1C*, *PDLIM5*, and *ACTA1*. To provide independent causal evidence, we performed siRNA-mediated knockdown of *Neat1* in NRVMs, which significantly downregulated *Pdlim5* and *Cacna1c* at both transcriptional and translational levels. Analysis of the GSE110209 dataset further revealed strong transcriptional correlations between *Neat1* and both *Cacna1c* and *Pdlim5* in the infarct border zone of rat hearts post-MI [40], supporting cross-species conservation of this regulatory network. Notably, this correlation was absent in sham-operated animals, indicating that the *NEAT1*–*CACNA1C* regulatory relationship is a specific consequence of the pathological MI microenvironment rather than a constitutive transcriptional program.

*NEAT1* has been reported to function as a microRNA sponge in some contexts [41]. Using Starbase and ENCORI, we identified *hsa-miR-204-5p/211-5p* as a shared candidate targeting *NEAT1*, *CACNA1C*, and *PDLIM5* [42,43], In AC16 cells, *NEAT1* knockdown significantly upregulated *hsa-miR-204-5p/211-5p* while downregulating both targets, a reciprocal pattern consistent with a ceRNA-mediated regulatory mechanism. Finally, to evaluate the therapeutic potential of *NEAT1* as a target, we performed Fluo-4 calcium imaging. Consistent with previous reports, 6 h of hypoxia markedly increased the Fluo-4 fluorescence intensity in NRVMs [44], indicative of an elevation in intracellular Ca^2+^ levels. siRNA-mediated knockdown of *NEAT1* effectively attenuated this elevation. *NEAT1* has been proposed as a potential therapeutic target in MI; previous in vivo studies have demonstrated that *NEAT1* silencing reduces infarct size, preserves cardiac function, and suppresses inflammatory and apoptotic signaling in mouse models of MI [45,46]. However, the relationship between *NEAT1* and *CACNA1C* is complex, although *NEAT1* knockdown downregulated *CACNA1C* transcription in vitro, *CACNA1C* regulation in vivo varies across species, time points, and molecular layers. Given this complexity, whether *NEAT1* knockdown exerts any net effect on Cav1.2 protein in the post-infarction border zone remains unclear. Meanwhile, genetic ablation of *NEAT1* has been reported to impair post-MI cardiac function in some settings, suggesting that the therapeutic outcome of *NEAT1* targeting may be context-dependent.

This study has several limitations that should be acknowledged. First, not all co-expression modules identified by hdWGCNA were subjected to in-depth investigation; and the border-zone-specific origin and electrophysiological properties of the aCM phenotype, which was defined primarily on a transcriptomic basis, still require further exploration. In addition, the aCM phenotype was defined primarily on the basis of transcriptomic data and has not been validated electrophysiologically. Second, owing to constraints inherent to the rodent model system [47], our functional studies focused primarily on the calcium channel gene *CACNA1C*, whereas the potassium channel gene *KCNQ1* was not experimentally pursued. Moreover, because the aCM phenotype derives from adult human border-zone myocardium, validation in NRVMs and AC16 cells may not fully recapitulate the adult ischemic context, which may limit translation to the human heart. Direct validation of the predicted miRNA–mRNA interactions, such as by dual-luciferase reporter assays, would further strengthen this mechanism. Third, although previous studies have reported beneficial effects of *NEAT1* knockdown in mouse models of MI, its electrophysiological consequences remain to be characterized. We focused on the susceptibility of individual cardiomyocytes as potential triggers of arrhythmia, but MEA recordings predominantly assess the tissue-level arrhythmic substrate rather than single-cell excitability, and patch-clamp recording was not feasible in our laboratory. Meanwhile, our Fluo-4 imaging captured static endpoint fluorescence intensity rather than dynamic Ca^2+^ transients, and stable in vitro recapitulation of the aCM phenotype remains challenging. Consequently, direct evidence from an in vivo arrhythmia model demonstrating the therapeutic efficacy of *NEAT1* inhibition is still lacking. In future work, we plan to establish a cardiac-specific *NEAT1* knockout mouse model using viral delivery of Cre recombinase to determine whether targeting *NEAT1* can reduce the incidence of malignant arrhythmias during the early phase of MI.

## 4. Materials and Methods

### 4.1. Single-Nucleus RNA Sequencing Data Collection and Processing

Single-nucleus RNA-seq (snRNA-seq) data were obtained from the previously published spatial multi-omic map of human myocardial infarction (Kuppe, Ramirez Flores, Li et al., 2022 [15]; Zenodo accession no. 6578047). The dataset comprises myocardial tissue specimens from patients with myocardial infarction and controls, profiled by snRNA-seq, snATAC-seq, and spatial transcriptomics (Visium). For the present study, we focused on the snRNA-seq component (snRNA-seq-submission.h5ad) and subset the cardiomyocyte (CM) nuclei for subpopulation-level re-analysis. Specifically, CM nuclei were re-embedded using uniform manifold approximation and projection (UMAP) with the first 30 harmony-corrected principal components. A shared nearest neighbour (SNN) graph was built using Seurat’s FindNeighbors, and nuclei were clustered with the Louvain algorithm (FindClusters) across multiple resolutions (0.1–1.5), with optimal resolution selection guided by cluster stability metrics visualized via clustree. Cluster-defining marker genes were identified by Wilcoxon rank-sum tests as implemented in Seurat’s FindAllMarkers. Based on the re-clustering results, we designated a distinct subpopulation (cluster 20) characterized by combined electrophysiological dysregulation and preserved metabolic activity signatures, which we designated arrhythmia-potential CM (aCM). The remaining CM nuclei retained their original annotation labels from the reference atlas, yielding four final CM subpopulations for downstream analyses: healthy CM (hCM), intermediate CM, damaged CM, and aCM.

### 4.2. Differential Expression and Gene Ontology Analysis

To characterize transcriptional differences between aCM and hCM, we performed differential expression analysis using Seurat’s FindMarkers function with the Wilcoxon rank-sum test. Differentially expressed genes (DEGs) were filtered by adjusted *p* < 0.05 and |log_2_ fold change| > 0.25. Gene Ontology (GO) enrichment analysis was performed using the DAVID Bioinformatics Resources (https://david.ncifcrf.gov/, https://davidbioinformatics.nih.gov/ (accessed on 12 June 2026)) on the filtered DEG list, with GO Biological Process (GO-BP) terms reported.

### 4.3. Gene Set Variation Analysis (GSVA) and Ucell

To evaluate pathway-level activity differences across CM subpopulations, we performed gene set variation analysis using the GSVA package (v1.50.0) with a Gaussian kernel. Pathway gene sets were compiled from three resources: (1) KEGG categories, (2) GO Biological Process terms from the Molecular Signatures Database (MSigDB, c5.go. bp v2025.1), and (3) Hallmark gene sets from MSigDB. GSVA enrichment scores were calculated on per-subpopulation pseudo-bulk expression profiles generated by AverageExpression. The resulting pathway activity matrix was visualized using pheatmap with row-wise z-score normalization and hierarchical clustering.

To test whether each CM cluster in a new, independent single-cell dataset of myocardial infarction recapitulates the four defined human CM subtypes (healthy, intermediate, damaged, arrhythmia-potential), we built four gene signatures and scored each cluster with UCell (AddModuleScore_UCell, an AUC-like enrichment score in [0, 1]; per-cell scores averaged per cluster). Markers per subtype came from differential expression, filtered at adjusted *p* < 0.05, |log2 fold change| > 0.25, and expression in >10% of CM cells. Signature genes were selected by a dominance criterion (higher mean expression in the target cluster(s) than in all others, ranked by dominance), each targeting the healthy, damaged, arrhythmia-susceptible, or transitional clusters, respectively. Score differences across clusters were tested by Kruskal–Wallis plus pairwise Wilcoxon (BH corrected) and visualized as zero-centered diverging heatmaps and dot plots combining relative point size with absolute score color.

### 4.4. Pseudotime Trajectory Analysis

To reconstruct the transcriptional trajectory of CM subclusters, we performed pseudotime analysis using Monocle 3. The Seurat object containing four CM subclusters (healthy, intermediate, damaged, and Arrhythmia_Susceptible_CM) was converted to a cell_data_set, preserving the pre-computed UMAP embedding. Cells were clustered at low resolution (1 × 10^−5^) and a principal graph was learned to capture the global differentiation topology. The trajectory was rooted at the healthy_CM population by selecting the most frequent graph node among healthy_CM cells. Branch-dependent genes were identified via the Moran’s I-based graph test (q < 0.05), and the top 500 most significant genes were hierarchically clustered (Ward’s D2) into four co-varying modules and visualized as a pseudotime-ordered heatmap after z-score normalization. To focus on the healthy_CM-to-Arrhythmia_Susceptible_CM transition, the shortest path between their representative graph nodes was extracted, and gene expression dynamics along this path were modeled by LOESS regression. Pseudotime-associated genes were identified by Spearman correlation (|ρ| > 0.3, adjusted *p* < 0.05) and LOESS goodness-of-fit (R^2^ > 0.05) along the path.

### 4.5. RNA Velocity and Pseudotime Analysis with scTour

To infer the transcriptional dynamics of CM subclusters, we performed RNA velocity-like analysis using scTour, a deep learning framework that simultaneously estimates pseudotime and a latent vector field without requiring spliced/unspliced count matrices. Raw UMI counts from the Arrhythmia_Susceptible_CM reference dataset were exported to AnnData format, preserving the pre-computed UMAP embedding from the Seurat workflow. Genes were filtered to the top 2000 highly variable genes (Seurat v3). The scTour model was trained for 400 epochs using a negative binomial loss, with reconstruction loss weights for the local and global components set to α_recon_lec = 0.5 and α_recon_lode = 0.5, respectively, and GPU acceleration was applied. Pseudotime was inferred using the get_time() function, and the latent transcriptional space (X_TNODE) was obtained by mixing the posterior and prior latent representations with mixing coefficients α_z = 0.5 and α_predz = 0.5. The vector field was computed from the latent space and then projected onto the pre-computed UMAP for visualization, which revealed the directionality and magnitude of transcriptional transitions across CM subclusters.

### 4.6. Spatial Transcriptomic Analysis

To determine the spatial distribution of aCMs in the post-MI heart, we analyzed Visium spatial transcriptomic data from four samples: one control (P1) and three MI samples spanning early (P2 IZ-BZ, ~5 days; P3 RZ-BZ, ~2 days) to late (P12 RZ-BZ, ~30 days) stages. Each spot was pre-annotated with cell-type proportions for 11 major cardiac cell types (RCTD deconvolution). Tissue zones were assigned based on cell-type composition: the infarct zone (IZ; cardiomyocyte < 15%, fibroblast + myeloid > 25%), remote zone (RZ; cardiomyocyte > 35%), and border zone (BZ; intermediate). To estimate CM subcluster proportions within each spot, we employed a gene signature scoring approach. Subcluster-specific marker genes were identified from the scRNA-seq CM reference using Wilcoxon rank-sum tests with Bonferroni correction (adjusted *p* < 0.01, log$_2$ fold change > 1.0), retaining the top 50 genes per subcluster that were also detected in Visium data. For each spot, a per-subcluster signature score was calculated as the mean expression of its marker genes across the four CM subclusters (healthy, intermediate, damaged, and Arrhythmia_Susceptible_CM), and scores were normalized to proportions via softmax transformation. Cell-type composition pie charts were overlaid onto H&E-stained tissue images at spot coordinates, and CM subcluster proportions were visualized as scatter plots with zone-level background shading. Log2 fold change relative to the control mean was computed to highlight regions of aCM enrichment or depletion.

### 4.7. Cell–Cell Communication Analysis

To characterize the intercellular signaling landscape involving aCMs, we performed ligand–receptor interaction analysis using CellChat (v2) with the full CellChatDB.human database. The scRNA-seq dataset was downsampled to a maximum of 1500 cells per cell type to balance computational efficiency and subtype representation. Communication probabilities were computed using the trimean method and filtered to retain interactions supported by at least 10 cells. To reduce dropout effects in shallowly sequenced single-cell data, expression values were smoothed via diffusion on the human protein–protein interaction network (projectData). Network centrality scores were calculated to identify major signal senders and receivers. To focus on aCM-specific signaling alterations, communication probabilities were aggregated at the pathway level separately by direction (sender/receiver) for each of the four CM subclusters (healthy, intermediate, damaged, and Arrhythmia_Susceptible_CM). Pathways were ranked by their total dispersion across CM subtypes (range of summed probability), and the top 12 most variable pathways were selected for visualization. Chord diagrams were used to display the global signaling architecture among all cell types, and dot plots were generated to compare pathway-level communication activity across the four CM subtypes.

### 4.8. hdWGCNA Analysis

To characterize the gene co-expression landscape of aCMs, we applied high-dimensional weighted gene co-expression network analysis (hdWGCNA) to aCM cells. Genes expressed in less than 5% of cells were excluded, retaining 11,724 genes. To mitigate sparsity, metacells were constructed by K-nearest neighbor aggregation (k = 25, max shared = 10) within each cell type–donor combination, using the harmony reduction. An unsigned co-expression network was then built with a soft-thresholding power of 3, yielding 11 modules (67–667 genes each; 3359 genes assigned in total). Module eigengenes were computed and harmonized to remove donor effects. Module eigengene activity was profiled across CM subclusters via DotPlot, in which M5 showed notably elevated expression and M10 showed depleted expression in aCMs compared to other CM subclusters, prompting their selection for downstream analysis. Module hub genes (top 10 by kME) were identified, and the transcriptional proximity of module gene sets was visualized by UMAP embedding (n_neighbors = 15, min_dist = 0.1). Genes from M5 and M10 were merged, and pairwise Pearson correlations were computed across metacells; edges with |r| > 0.75 were retained and used to construct a co-expression network via igraph, which was subsequently exported to Cytoscape (Version 3.9.1) for visualization and network analysis.

### 4.9. In Silico Knockout

For scTenifoldNet, we simulated *NEAT1* perturbation using the single-cell Tensor-based Network Knockout framework. Co-expression networks were constructed from bootstrap samples (10 sub-networks, 500 cells each) of the Arrhythmia_Susceptible_CM expression matrix, with genes filtered at the 90th percentile of expression variance. Network topology was decomposed into 10 core regulatory patterns via tensor CP factorization (1000 iterations), and wild-type versus knockout networks were aligned in a 10-dimensional manifold space. For each gene, differential regulation was quantified by the Z-score of its inter-network distance, with significance assessed by adjusted *p* value derived from the tensor decomposition. For GenKI, we adopted a variational graph auto-encoder (VGAE)-based framework to infer the downstream transcriptional consequences of *NEAT1* depletion. A gene co-expression network was first constructed from scRNA-seq data of Arrhythmia_Susceptible_CM cells (*n* = 1264) using pcNet. The VGAE was subsequently trained on wild-type data to learn graph-structured latent representations, and *NEAT1* knockout was simulated by zeroing its expression and removing all associated edges from the network. For each gene, the KL divergence between wild-type and knockout latent distributions was calculated, with significance assessed via permutation testing (100 iterations).

### 4.10. Neonatal Rat Ventricular Myocytes Isolation and Culture

Neonatal rat ventricular myocytes (NRVMs) were isolated using the Worthington Neonatal Cardiomyocyte Isolation System (WBC-LK003300) (Hangzhou Ziyuan Experimental Animals Technology Co., Ltd.). Briefly, hearts from anesthetized rat pups were excised, rinsed in ice-cold Sterile calcium- and magnesium-free Hank’s Balanced Salt Solution, and minced to <1 mm^3^ fragments. Tissue was digested overnight at 4 °C with 50 µg/mL trypsin, followed by 30–45 min collagenase treatment at 37 °C with intermittent trituration. Cell suspensions were sequentially filtered, centrifuged (1200× *g*, 5 min), and subjected to RBC lysis. Cardiomyocytes were enriched via 1.5 h differential adhesion, then cultured in 10% FBS/DMEM with antibiotics on gelatin-coated plates (37 °C, 5% CO_2_). To induce hypoxia, NRVMs were placed in a hypoxia chamber (1% O_2_, 5% CO_2_, 94% N_2_) for 6 h. The human cardiomyocyte cell line AC16 was cultured under identical conditions.

### 4.11. Cell Culture and siRNA Transfection

AC16 human cardiomyocytes and NRVMs were cultured under standard conditions. For NEAT1 knockdown, cells were transfected with specific siRNAs targeting *NEAT1* (sequences listed in Table S2) using Lipofectamine™ RNAiMAX Transfection Reagent (Invitrogen, Carlsbad, CA, USA) according to the manufacturer’s protocol. Cells were harvested 24 h post-transfection for subsequent analyses.

### 4.12. Western Blot

Western blot was used for immunoblotting analysis. The extracted proteins were separated by SDS-PAGE and transferred to a 0.22 μm PVDF membrane. The membrane was sequentially probed with primary antibodies and HRP-conjugated secondary antibodies, followed by detection using enhanced chemiluminescent substrate. For CACNA1C, a multi-pass transmembrane protein, protein samples were not boiled prior to loading.

### 4.13. RT-qPCR

Real-time quantitative PCR was performed using SYBR Master Mix (Vazyme, Nanjing, China) on a BIO-RAD CFX96 Real-Time System (Bio-Rad Laboratories; Hercules, CA, USA). Each reaction was run in triplicate, and relative gene expression was calculated by the 2^−ΔΔCt^ method with β-tubulin as the endogenous control. Primer sequences for rat (NRVM) and human (AC16) genes are listed in Table S2. For miRNA quantification, total RNA was reverse-transcribed using the miRcute Enhanced miRNA cDNA First Strand Synthesis Kit (KR211, Tiangen, Beijing, China), and qPCR was performed with the miRcute Enhanced miRNA qPCR Detection Kit (SYBR Green, FP411, Tiangen) on the same instrument.

### 4.14. Fluo-4 Calcium Imaging

After 6 h of hypoxia, NRVMs were incubated with 5 µM Fluo-4 AM (Yeasen, China) in HBSS at 37 °C for 30 min in the dark, followed by three washes with HBSS and an additional 20 min incubation at 37 °C to allow for complete de-esterification. Fluorescence images were captured under an inverted fluorescence microscope (Olympus IX83, Japan) with excitation at 488 nm and emission at 516 nm. One field per well was recorded from five independent wells of a 24-well plate, and mean fluorescence intensity was quantified using ImageJ (1.54p) after background subtraction. Relative intracellular calcium levels were normalized to the normoxia control group. Data are presented as mean ± SD.

### 4.15. Statistical Analysis

Statistical analyses were performed using GraphPad Prism 8 (GraphPad Software, La Jolla, CA, USA). Each data point represents an independent biological replicate. Comparisons between two groups were performed using an unpaired two-tailed Student’s *t*-test. Comparisons among multiple groups (e.g., Figure 1F and Figure 5E) were performed using one-way analysis of variance (ANOVA) followed by Bonferroni’s post hoc test for multiple comparisons. Statistical significance was defined as *p* < 0.05.

## 5. Conclusions

Cardiomyocytes in the early MI border zone exhibit ion channel remodeling driven by elevated *NEAT1*, which potentially modulates *CACNA1C* and *PDLIM5* via a microRNA-mediated ceRNA network. These findings suggest that *NEAT1* warrants further investigation as a potential contributor to malignant arrhythmias in early-stage MI.

## Data Availability

The snRNA-seq data used for the main analysis are openly available in the Zenodo repository under accession number 6578047 (Kuppe, Ramirez Flores, Li et al., 2022) [15] and can be accessed at https://zenodo.org/record/6578047 (accessed on 24 March 2026). The external validation dataset (GSE110209 and Zenodo 17410821) is publicly available in the Gene Expression Omnibus (GEO) at https://www.ncbi.nlm.nih.gov/geo/query/acc.cgi?acc=GSE110209 (accessed on 12 April 2026) and Zenodo at https://zenodo.org/records/17410821 (accessed on 12 April 2026). No new sequencing data were generated in this study. All analysis code required to reproduce the results has been uploaded to the GitHub repository: https://github.com/qianqiandit/NEAT1-coordinates-a-PDLIM5-CACNA1C-regulatory-program.

## Supplementary Materials

The following supporting information can be downloaded at: https://www.mdpi.com/article/10.3390/ijms27177945/s1.

## Abbreviations

The following abbreviations are used in this manuscript:

aCM	arrhythmia-susceptible cardiomyocyte
ceRNA	competing endogenous RNA
CM	cardiomyocytes
dCM	damaged cardiomyocyte
DEGs	differentially expressed genes
GO	Gene Ontology
GSVA	gene set variation analysis
hCM	healthy cardiomyocyte
iCM	intermediate cardiomyocyte
NRVM	neonatal rat ventricular myocyte
snRNA-seq	single-nucleus RNA sequencing
