# Supplementary material for: NEAT1 Coordinates a PDLIM5–CACNA1C Regulatory Program Associated with a Potentially Arrhythmogenic Cardiomyocyte State in the Border Zone During Early Myocardial Infarction
Source: Int J Mol Sci. 2026 Sep 7;27(17):7945. doi: 10.3390/ijms27177945 (PMC13565834; doi:10.3390/ijms27177945)

Supplementary Figure S2

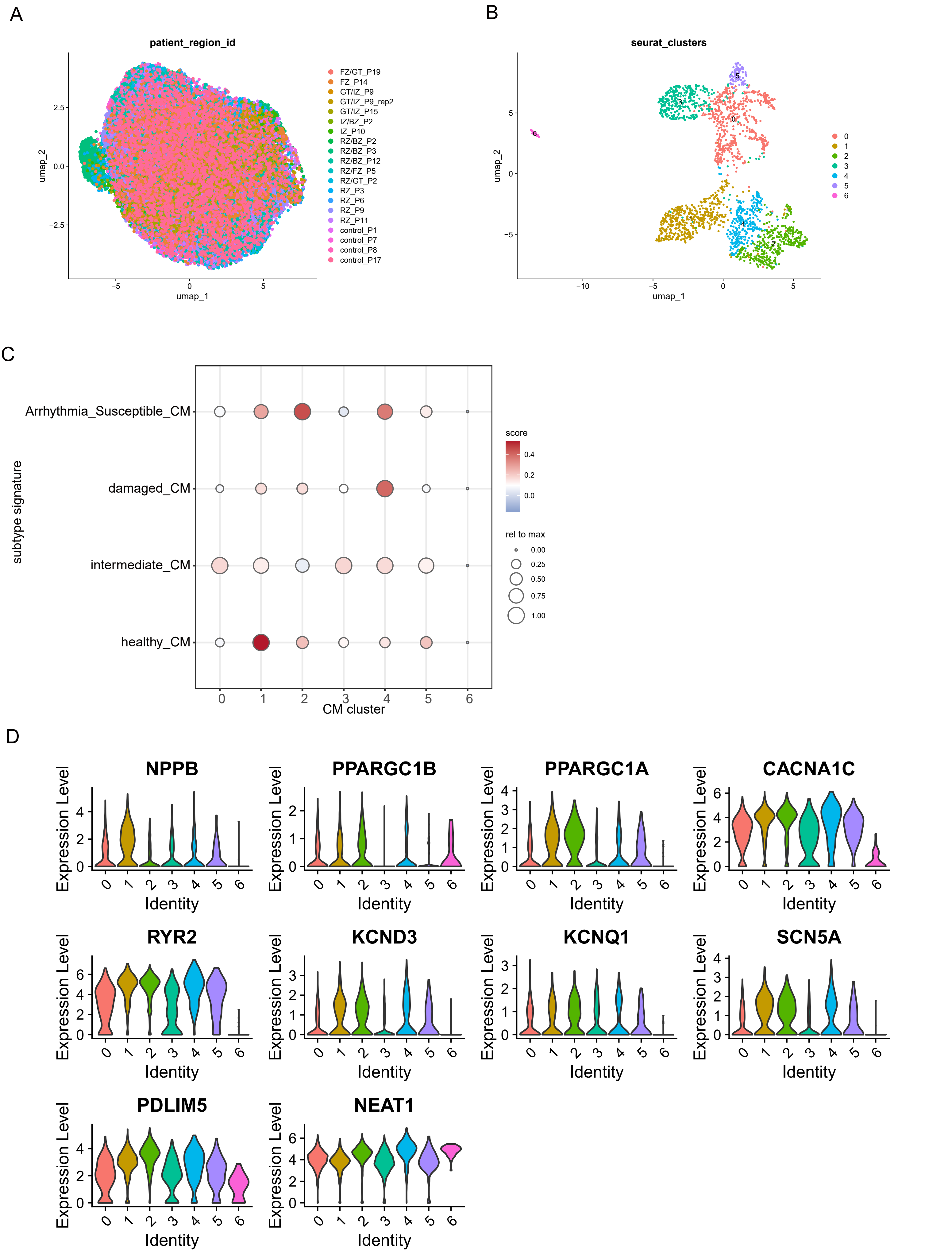

Supplement: Supplementary file 1 [file ijms-27-07945-s001.zip › sup_figS2.pdf]
